# Developmental Characteristics of Skeletal Muscle during the Embryonic Stage in Chinese Yellow Quail (*Coturnix japonica*)

**DOI:** 10.3390/ani13142317

**Published:** 2023-07-14

**Authors:** Li Liu, Lingqian Yin, Yaohan Yuan, Yuan Tang, Zhongzhen Lin, Yiping Liu, Jiandong Yang

**Affiliations:** 1 Key Laboratory of Livestock and Poultry Multi-Omics, Ministry of Agriculture and Rural Affairs, College of Animal Science and Technology, Sichuan Agricultural University, Chengdu 611130, China; 2 Farm Animal Genetic Resources Exploration and Innovation Key Laboratory of Sichuan Province, Sichuan Agricultural University, Chengdu 611130, China

**Keywords:** quail, embryo, myogenesis, myofiber, mRNA expression

## Abstract

**Simple Summary:**

The current work revealed the developmental characteristics of skeletal muscle and the expression patterns of related regulatory genes in the embryonic stage of quails. During the embryonic stage, the weight and fiber size of the leg muscle was larger than the breast muscle. The *MyoD* and *Pax7* genes were critical myogenic regulatory factors and were highly expressed in the middle stage of the embryonic period in breast and leg muscles. Overall, these results imply that day 12 of quail embryos may be a crucial point for muscle development.

**Abstract:**

The quail is an important research model, and the demand for quail meat has been increasing in recent years; therefore, it is worthwhile investigating the development of embryonic skeletal muscle and the expression patterns of regulatory genes. In this study, the expression of *MyoD* and *Pax7* in the breast muscle (*m. pectoralis major*) and leg muscle (*m. biceps femoris*) of quail embryos on days 10 through 17 were determined using qRT-PCR. Paraffin sections of embryonic muscle were analyzed to characterize changes over time. Results showed that *MyoD* and *Pax7* were expressed in both breast and leg muscles and played a significant role in embryonic muscle development. Compared to breast muscle, leg muscle grew faster and had greater weight and myofiber size. The findings suggested that embryonic day 12 (E12) may be a key point for muscle development. Correlation analysis showed that *MyoD* expression was significantly negatively correlated with muscle and embryo weight, whereas *Pax7* gene expression had no significant correlation with these characteristics. These fundamental results provide a theoretical basis for understanding the characteristics and transition points of skeletal muscle development in quail embryos and an important reference for farmers raising quail from eggs.

## 1. Introduction

In poultry raised for meat, the main edible portion is concentrated in skeletal muscle; hence, optimal muscle development is essential for good commercial production of animals. Myogenesis is a complex process that can be divided into two phases: embryonic and postnatal muscle development. Several studies showed that myofiber number was usually determined at the embryonic stage, whereas muscle growth mainly depended on the hypertrophy of muscle fibers after birth. During the embryonic stage, myogenic progenitor cells proliferate and differentiate extensively, and myoblasts are then fused into multinucleated myotubes and myofibers [1]. Muscle cells grow and mature until postnatal. After hatching, the growth of muscle is characterized by the increase in length and diameter of myofibers [2]. The quail (*Coturnix japonica*) belongs to the Phasianidae of the Galliformes and is widely raised because of its relatively short incubation period (about 17 days), convenient feeding, rapid growth, early maturation, and excellent production properties [3]. As a small poultry breed, quail is mainly used for meat and egg production. Although quail eggs are more popular than quail meat [4], quail is still considered a good source of meat products [5], especially in developing countries such as South Africa [6]. In addition, with the diversification of quail meat processing methods and new products [7,8], quail meat is also increasingly being chosen by consumers.

During the embryonic stage, it has been reported that myogenesis is tightly regulated by the expression of numerous genes, such as paired box 7 (*Pax7*) and myogenic regulatory factors (MRFs), including myogenic factor 5 (*Myf5*) [9], myogenic differentiation factor (*MyoD*) [10], myogenin (*MyoG*) [11], and myogenic regulatory factor 4 (*MRF4*) [12]. Among the above factors, the MRFs constitute the regulatory center and play a key role in regulating the proliferation and differentiation of myoblasts, as well as the number and size of muscle fibers. *MyoD* was the first of the MRF family to be identified [13]. As a major regulator of myoblast differentiation, *MyoD* is important in the proliferation of myogenic progenitor cells and in the terminal differentiation of myoblasts [14]. It has been reported that silencing this factor inhibited sarcomere formation and further muscle formation [15]. In the absence of both *MyoD* and *Myf5*, there was a complete deficiency of myoblasts and muscle fibers [16]. These results indicate that *MyoD* is essential for the formation of embryonic muscle.

*Pax7* is a member of the paired-box protein family, which plays an essential role in skeletal muscle development and muscle regeneration [17,18,19]. In previous studies, it has been found that silencing *Pax7* could lead to the death of mice in the early postnatal stage [20]. It was confirmed that a lack of *Pax3* and *Pax7* interrupted muscle development; only the early embryonic muscle of the myotome was formed [21]. These findings demonstrated that *Pax7* also plays a crucial role in embryonic muscle development.

*MyoD* and *Pax7* are considered candidate genes for enhancing meat production in poultry. However, there is currently a lack of reports on the expression of *MyoD* and *Pax7* in quail and their correlation with muscle development, particularly in the leg muscles. Although quail is frequently used as an animal model for muscle development [22,23], there have been no published studies on the histochemical characteristics of its embryonic development at different stages. Thus, the aim of this study was to reveal the developmental characteristics of the skeletal muscle and the expression patterns of related regulatory genes during embryogenesis in quail. Specifically, we measured the expression pattern of *MyoD* and *Pax7* in the breast and leg muscles of quail embryos from days 10 to 17 and recorded the morphologic characteristics of muscle fiber. Subsequently, further analysis was conducted on the correlation between the expression of these two genes and muscle development. This study will provide reference information for poultry breeding and a description of the characteristics of skeletal muscle development during the embryonic stage.

## 2. Material and Methods

### 2.1. Sample Collection

A total of 500 fertilized eggs were collected from the same population of Chinese Yellow Quail (*Coturnix japonica*) provided by Yunge Quail Professional Cooperative of Dongpo District (Meishan, China). Before being placed in an automatic incubator, all eggs were disinfected with 30% hydrogen peroxide by spraying [24]. These fresh eggs were incubated under the same conditions at a temperature of 37.8 ± 0.5 °C and humidity of 60–70%. The eggs were turned every two hours during the incubation period. The quail embryos were randomly picked out from days 10 to 17 (*n* = 6 per day), respectively. For each embryo, the left breast and leg muscles were dissected from the body, immediately frozen in liquid nitrogen, and stored at −80 °C for total RNA extraction (*n* = 3 per day), and the right side was used for morphological observation (*n* = 3 per day). Meanwhile, during the sampling period, ten quail embryos were randomly selected, and the weight of embryos, entire breast, and leg muscles were measured. All embryo experiments in this study were approved by Sichuan Agricultural University’s Animal Care and Use Committee with the approval number 2021202052.

### 2.2. Morphometric Analysis

One piece of meat (1 × 0.5 × 0.5) was taken from the *m. pectoralis major* of the breast muscle and the *m. biceps femoris* of the leg muscle. All samples obtained were cut by the surgical blade perpendicular to the direction of muscle fibers. The samples were initially fixed in 4% paraformaldehyde for 48 h, followed by a series of graded alcohol dehydration and paraffin-embedded. Subsequently, the samples were sliced into a thickness of 5 µm and stained with hematoxylin and eosin. The micrographs were taken by a digital microscope (Nikon SI, Tokyo, Japan) and analyzed using Image-Pro Plus Version 6.0 software (Media Cybernetics, Inc., Rockville, MD, USA). Pictures were magnified 200 times to measure muscle fiber diameter (µm), muscle fiber cross-sectional area (µm^2^), and muscle fiber density (N/mm^2^). The muscle fiber cross-sectional area of each sample was measured by randomly selecting 30 intact and well-oriented muscle fibers. After obtaining the muscle fiber cross-sectional area (A), we regarded muscle fibers as round and calculated their diameter (D) using the formula D = 2√A/π [25]. Thereafter, the muscle fiber density (d) was calculated by selecting the total area of the visual field (S) and the corresponding number of muscle fibers (N), and the formula was d = N/S [26]. All fields in each section were selected randomly.

### 2.3. RNA Extraction, Reverse Transcription, and Quantitative Real-Time PCR

Total RNA was extracted from the above muscle tissues of quails using Trizol (TaKaRa, Dalian, China) following the instructions. The RNA concentration and purity were assessed by NanoDrop 2000C spectrophotometer (Thermo, San Jose, CA, USA). The reverse transcription was performed according to the instructions of the PrimeScript RT Reagent Kit (TaKaRa, Dalian, China). The primer of *MyoD* and *Pax7*, and *GAPDH* (the reference gene) were designed by using Prime Premier 6.0 (PREMIER Biosoft, San Francisco, CA, USA) (Table 1). Each reaction (10 μL) consisted of 5 μL TB Green^®^ Premix Ex Taq II, 0.5 µL each primer, 1 µL cDNA, and 3 µL ddH_2_O. The reaction condition for real-time PCR was as follows: initial denaturation at 95 °C for 3 min, followed by 40 cycles of 95 °C for 10 s, annealing for 57–60 °C for 20 s, and extension at 72 °C for 20 s. There were three replicates for each sample, and the relative expression levels were calculated by the 2^−∆∆Ct^ method.

### 2.4. Statistical Analysis

The statistical analysis was performed using SPSS 27.0 (SPSS, Inc., Chicago, IL, USA). The data corresponding to the normal distribution were analyzed using one-way ANOVA or Student’s *t*-test for the comparison of the mean among different groups. The Student–Newman–Keuls test was used for multiple comparisons. Additionally, bivariate correlation was used to evaluate the correlation between the expression of *MyoD*, *Pax7,* and muscle characteristics. The significant levels were regarded as *p* < 0.05 and *p* < 0.01, and the data were expressed as means ± standard error.

## 3. Results

### 3.1. Weight of Embryos, Breast, and Leg Muscles

**Figure 1:**
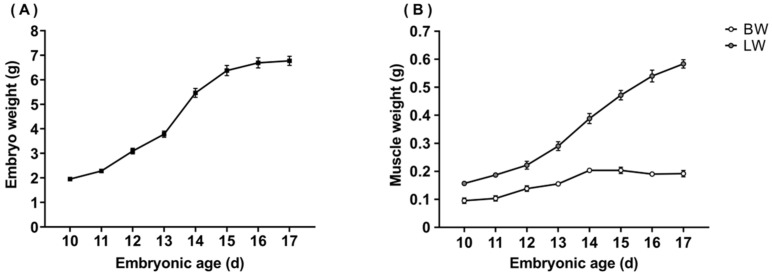
Embryo weight, breast weight (BW), and leg weight (LW) at each embryonic age. (**A**) embryo weight, (**B**) breast weight and leg weight.

The weight of the embryo, breast, and leg muscles of quails at each embryonic age are presented in Figure 1. The weight of embryo, breast, and leg muscles showed an upward trend with the increasing of the embryonic age. The maximal growth rate of embryo and muscle both occurred between 13 and 14 days, followed by days 11 to 12. In addition, the growth rate of the leg muscle was much higher than that of the breast muscle from the middle to the late embryonic stage.

### 3.2. Histological Morphology of Breast and Leg Muscles

**Figure 2:**
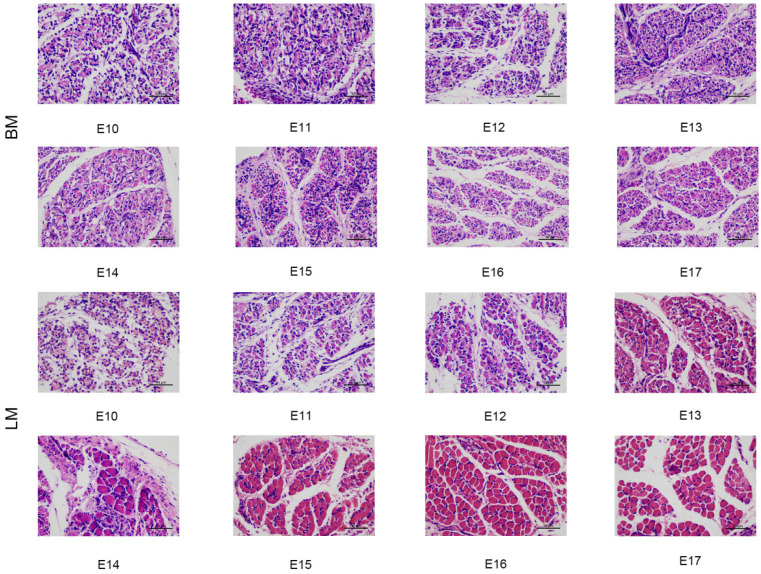
The photomicrographs of paraffin sections of breast and leg muscles of quails on embryonic day 10 (E10), 11 (E11), 12 (E12), 13 (E13), 14 (E14), 15 (E15), 16 (E16), and 17 (E17). The slices were all made under the same conditions (H&E, 400×).

The photomicrographs of breast and leg muscles in quail embryos from days 10 to 17 are shown in Figure 2. The myotube thickened and adhered to form a myofiber bundle from day 10 to day 11. The number of free myoblasts around the myotube was gradually reduced. The muscle fibers could be observed obviously on embryonic day 12. During the subsequent development of the embryo, there were few free muscle cells, and muscle fibers continued to develop until embryonic day 17. Compared to the leg muscle, the myofiber borders of breast muscles were difficult to identify on embryonic day 10.

### 3.3. Morphometric Analysis

**Figure 3:**
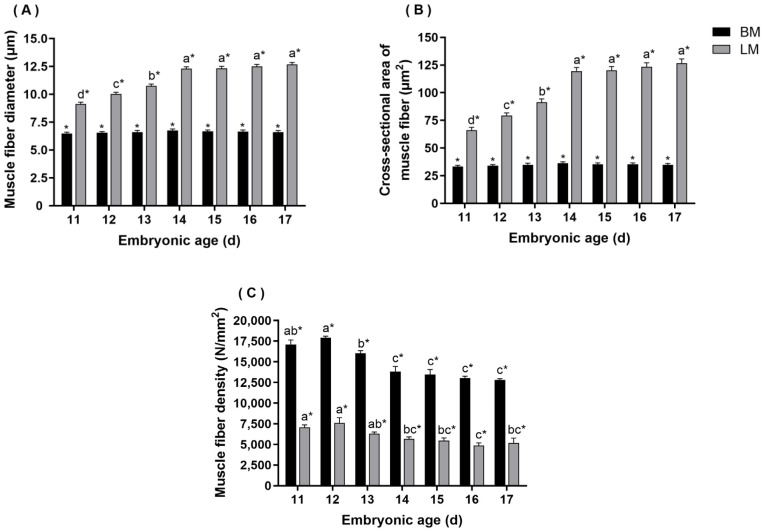
The diameter, cross-sectional area, and density of muscle fibers in breast and leg muscles of quails. (**A**) diameter, (**B**) cross-sectional area, (**C**) density. Different lowercase letters denoted that the difference was significant among different embryonic ages for the same tissue (*p* < 0.05). A superscript asterisk (*) indicates that the difference was significant between different tissue on the same embryonic day (*p* < 0.05), and no asterisk indicates no significant difference.

Due to the difficulty in distinguishing breast muscle fibers on embryonic day 10, the results of the diameter, cross-sectional area, and density of muscle fibers in skeletal muscle were measured from days 11 to 17. As shown in Figure 3, it could be observed that the diameter and cross-sectional area of muscle fibers in breast muscle increased from day 11, reaching the peak on embryonic day 14, followed by a downward trend. Throughout the test cycle, myofiber diameter and cross-sectional area in leg muscle were gradually increased with the embryo developed continually. Additionally, the density of muscle fibers in breast and leg muscles increased slightly from days 11 to 12 and then showed a downward trend until the time before hatching (E17). It should be noted that there were significant differences in diameter, cross-sectional area, and density between the breast muscle and the leg muscle at each embryonic age (*p* < 0.05).

### 3.4. The Expression Pattern of MyoD in Breast and Leg Muscle Tissues

**Figure 4:**
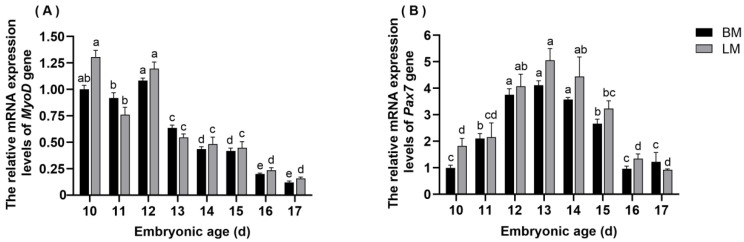
The expression levels of *MyoD* and *Pax7* in the breast and leg muscles of quail embryos. (**A**) *MyoD* mRNA, (**B**) *Pax7* mRNA. Different lowercase letters denoted that the difference was significant among different embryonic ages for the same tissue (*p* < 0.05).

The mRNA expression levels of *MyoD* in quail embryonic skeletal muscle are shown in Figure 4A. The expression trend of *MyoD* in breast and leg muscle tissues of quail embryos was consistent. There was no significant difference in the expression levels of *MyoD* between the breast muscle and the leg muscle at each embryonic age. In the breast and leg muscles of quail, the mRNA expression levels of *MyoD* were significantly higher on embryonic days 10, 11, and 12 compared with the other days (*p* < 0.05). In the later stages, the expression of *MyoD* decreased gradually and maintained at lower levels.

### 3.5. The Expression Pattern of Pax7 in Breast and Leg Muscle Tissues

The expression pattern of *Pax7* mRNA in quail embryonic skeletal muscle is shown in Figure 4B. The results indicated that the expression levels of *Pax7* in both kinds of muscles showed a trend of increasing and then decreasing. It was worth mentioning that there was no significant difference in the expression of *Pax7* between the breast muscle and the leg muscle at each embryonic age. The expression of *Pax7* was higher on embryonic days 12, 13, and 14 in the breast muscles than on the other days (*p* < 0.05), while it was higher in leg muscles from days 12 to 15 (*p* < 0.05). Meanwhile, the peak was obtained on embryonic day 13, both in the breast and leg muscles of quail embryos.

### 3.6. Correlation Analysis in Different Characteristics

As shown in Table 2, the results showed that the expression of *MyoD* was negatively correlated with myofiber diameter, cross-sectional area, breast weight, and embryo weight, whereas it was significantly positively correlated with muscle fiber density (*p* < 0.01). There was no significant correlation between the expression level of *Pax7* and the above characteristics. The diameter of muscle fibers was significantly positively correlated with the cross-sectional area of myofibers (*p* < 0.01). Similar to the diameter of muscle fibers, the cross-sectional area of muscle fibers was significantly positively correlated with breast weight (*p* < 0.01). The density of muscle fibers was significantly positively correlated with breast weight and embryo weight (*p* < 0.01) and negatively correlated with diameter and cross-sectional area.

The correlation coefficients between the expression level of *MyoD*, *Pax7,* and myofiber characteristics, leg weight, and embryo weight are summarized in Table 3. The results revealed that the expression levels of *MyoD* were positively correlated with the density of muscle fiber (*p* < 0.01) and negatively correlated with the diameter (*p* < 0.05), cross-sectional area (*p* < 0.05), leg weight (*p* < 0.05), and embryo weight (*p* < 0.05). The diameter of muscle fibers was positively correlated with the muscle fiber cross-sectional area (*p* < 0.01), leg weight (*p* < 0.01), and embryo weight (*p* < 0.01). Moreover, the cross-sectional area of muscle fiber was positively correlated with leg weight (*p* < 0.01) and embryo weight (*p* < 0.01). The density of muscle fiber was negatively correlated with a diameter (*p* < 0.01), cross-sectional area (*p* < 0.01), leg weight (*p* < 0.01), and embryo weight (*p* < 0.01).

## 4. Discussion

The regulation of the growth and development of skeletal muscle has recently received much attention among researchers. The embryonic stage is especially important for subsequent animal growth and development. In this research, the changes in the weight of the breast and leg muscles and the embryo weight from days 10 to 17 were measured. The results showed a trend towards sustained increase except for breast weight. We found that the proportion of leg muscle mass to embryonic size and the growth rate of leg muscle was greater than that of breast muscle. As an early maturing species, the chicks of quail rely on relatively mature leg muscles to support walking, foraging, and generating heat before flying; therefore, more nutrients are allocated to the development of leg muscles during the embryonic stage to support their faster development [27,28].

During the embryonic stage, myoblasts proliferate, fuse, and finally form into muscle fibers. In this process, the morphological structure and quantity of myofibers are completely determined, which has a direct impact on the growth and development of postnatal muscle [29,30,31]. Based on the results of a histomorphological examination of the breast and leg muscle of quails during the embryonic stages, the muscle fibers were obvious by embryonic day 12 in our study. Zhu et al. also found that mature muscle fiber bundles were present at this time [32]. These findings support the hypothesis that muscle fibers are basically mature on embryonic day 12. On the 10th day of embryo formation, it was difficult to distinguish the breast muscle fibers compared to the leg muscles, further confirming that the leg muscle developed faster than the breast muscle.

The characteristics of muscle fiber are commonly used as essential parameters to evaluate meat quantity and quality during growth and development [33,34,35]. It has been reported that sex had no effect on muscle fiber characteristics [36,37]. Therefore, this study did not separately investigate the effects of gender. In our results, the diameter and cross-sectional area of muscle fibers in the leg muscle gradually increased as the embryo developed. However, myofiber diameter and cross-sectional area in breast muscle increased from days 11 to 14 and showed a slight decrease after day 14, which is consistent with the results demonstrated by Zhu et al. [32]. In the late stages of embryo development, the yolk nutrients cannot supply the entire energy needs of avian embryos, so skeletal muscle proteins were mobilized to ensure an energy supply. The diameter, area, and quantity of muscle fibers were correspondingly reduced, further reducing the weight of skeletal muscle, especially in the breast, which contains more IIb-type muscle fibers [38,39,40]. This might be the reason for the slight decrease in myofiber diameter and cross-sectional area of the breast muscle before hatching. In addition, the myofiber density of breast and leg muscles increased slightly from days 11 to 12 and then showed a downward trend. Similar results were obtained in studies of ducks, where the average density constantly decreased from E21 (embryonic day 21) to E27 (embryonic day 27), indicating that the number of muscle fibers continued to increase from the early to the middle stages of embryonic development, reaching a peak around E21, and then remained relatively fixed [37]. In quail embryos, we found that the number of muscle fibers reached its peak and was basically fixed on embryonic day 12.

Muscle development during the embryonic period is an extremely complex process reflected in the precise regulation of muscle development-related genes at different time points [41]. As a member of the MRF gene family, *MyoD* plays a key role in promoting proliferation in growing myoblasts and specifying myogenic progenitors. It has been reported that silencing *MyoD* expression leads to muscle formation failure. In the present study, the expression levels of *MyoD* in the breast and leg muscles of quail embryos from day 10 to day 17 were examined. In the breast and leg muscles, the expression of *MyoD* was higher on days 10, 11, and 12 and then rapidly decreased until birth, which is in good agreement with previous findings [42]. These results further support the hypothesis that embryonic day 12 might be a key point in quail muscle development. Li et al. found that the *MyoD* mRNA was expressed in both leg and breast muscles during the embryonic stage of ducks, and the expression profile showed a similar trend, while the expression level of *MyoD* in breast muscle was significantly higher than that in leg muscle at each embryonic age [43]. In contrast, our study showed that *MyoD* mRNA expression was lower in the breast muscle, but the difference between the breast and leg muscles was not significant. These results showed that the expression of *MyoD* varied among different species, but its crucial role in the growth and development of embryonic skeletal muscle cannot be denied.

Reports have confirmed that the absence of *Pax7* affected the formation of muscle, indicating the importance of this gene in muscle growth. In the current research, *Pax7* mRNA was expressed from days 11 to 17, but the expression level was not significantly different between breast and leg muscles. We also found higher expression of *Pax7* in both kinds of muscle tissue on days 12, 13, and 14 of the embryonic stage (*p* < 0.05). The pattern of *Pax7* expression can be ascribed to the fact that the muscle progenitor cell population already existed in the embryonic stage. In birds, secondary myogenesis takes place after embryonic day 6 [44]. Messina et al. reported that skeletal muscle progenitor cells can differentiate into a large number of muscle fibers during secondary myogenesis under the regulation of *Pax7* [45]. When the muscle matured, these progenitor cells transitioned to a quiescent state, becoming known as satellite cells [46]. According to reports in mice, after a second wave of myogenesis in 14.5- to 17.5-day embryos, satellite cells appearing as monocytes could be recognized between the muscle fiber plasmalemma and the basement membrane [45]. These satellite cells also expressed *Pax7* and remained in the muscle as a reserve population to mediate the postnatal growth of skeletal muscle [47]. As reported by Wang et al., the muscle mass of adult Beijing ducks was higher than that of Jianchang ducks and Haiwu ducks, which correspond to a large number of satellite cells [46]. In light of the results of our study and previous studies, it has been shown that *Pax7* makes a significant contribution to the growth and development of muscle before and after birth.

The results of correlation analysis showed that *MyoD* gene expression in both kinds of muscles was negatively correlated with muscle fiber size, embryo weight, and weight of skeletal muscle. Furthermore, the correlation between *MyoD* gene expression and leg weight was higher than that with breast muscle, which is consistent with the study carried out by Wang et al. in the embryonic stage of ducks [48]. However, *Pax7* expression showed no significant correlation with all characteristics in our study. Yin et al. found that compared to low-weight lines of chickens, *Pax7* expression levels in the pectoralis major muscle of high-weight lines of chickens were lower on hatching day and higher on the 28th day after birth [49]. These results suggest that *Pax7* may play a more prominent role in hypertrophy and weight gain of muscle postpartum compared with the embryonic stage. In addition, the results showed that the diameter and cross-sectional area of muscle fibers were positively correlated with embryo weight, while the density of muscle fibers was negatively correlated with embryo weight, indicating a coordinated regulation of muscle fiber development and weight gain in quails.

## 5. Conclusions

Based on this study, we conclude that the growth rate of leg muscle in quail embryos was higher than that of breast muscle. Both *MyoD* and *Pax7* mRNA were expressed in the breast and leg muscles of quail embryos and played an important role in embryonic muscle development. Furthermore, we infer that embryonic day 12 may be a key point for muscle development. These results contribute to our understanding of the developmental processes and the transition points of quail skeletal muscles during the embryonic stage. The specific mechanisms by which *MyoD* and *Pax7* genes regulate muscle development need further research and verification. Experiments should be conducted to confirm the formation of transition periods in quail skeletal muscle development through mRNA transcription profiles and protein expression levels of other key genes involved in muscle development.

## Figures and Tables

**Table 1:** The primer sequence information of *MyoD* and *Pax7* for RT-PCR analysis.

Gene Symbol	Primer Sequence (5′→3′)	Product Length (bp)	Annealing Temperature (°C)	Accession No.
*MyoD*	F: ACTACAGCGGGGAGTCAGAT	149	57	NM_204214.3
	R: CCCATGCTTTGGGTCATTTGG			
*Pax7*	F: TCGATTAGCCGTGTGCTACG	108	59.2	NM_205065.1
	R: GCCATCTATGCTGTGCTTGG			
*GAPDH*	F: GGGGAAAGTCATCCCTGAGC	145	60	NM_204305.1
	R: AGCAGCCTTCACTACCCTCT			

F: Forward primer; R: Reverse primer.

**Table 2:** The correlation coefficients (r) between the expression level of *MyoD*, *Pax7*, and other characteristics of breast muscles during embryonic development from days 11 to 17.

Variable	Diameter (µm)	Cross-Sectional Area (µm^2^)	Density (N/mm^2^)	Breast Weight (g)	Embryo Weight (g)
*MyoD*	−0.601	−0.638	0.976 **	−0.822 *	−0.936 **
*Pax7*	0.136	0.120	0.564	−0.188	−0.528
Diameter (µm)		0.997 **	−0.687	0.898 **	0.690
Cross-sectional area (µm^2^)			−0.711	0.904 **	0.706
Density (N/mm^2^)				−0.887 **	−0.965 **

The superscript asterisk (*) and double asterisk (**) indicate a significant correlation at the level of *p* < 0.05 and *p* < 0.01, respectively.

**Table 3:** The correlation coefficients (r) between the expression level of *MyoD*, *Pax7,* and other characteristics of leg muscles during embryonic development from days 11 to 17.

Variable	Diameter (µm)	Cross-Sectional Area (µm^2^)	Density (N/mm^2^)	Leg Weight (g)	Embryo Weight (g)
*MyoD*	−0.799 *	−0.811 *	0.952 **	−0.866 *	−0.825 *
*Pax7*	−0.284	−0.314	0.453	−0.564	−0.435
Diameter (µm)		0.999 **	−0.923 **	0.941 **	0.981 **
Cross-sectional area (µm^2^)			−0.930 **	0.949 **	0.985 **
Density (N/mm^2^)				−0.939 **	−0.940 **

The superscript asterisk (*) and double asterisk (**) indicate a significant correlation at the level of *p* < 0.05 and *p* < 0.01, respectively.

## Data Availability

The data relevant to the study are available from the corresponding author upon reasonable request.
